# Minimally invasive unilateral versus bilateral technique in performing single-segment pedicle screw fixation and lumbar interbody fusion

**DOI:** 10.1186/s13018-015-0253-1

**Published:** 2015-07-16

**Authors:** Chen Chen, Xuecheng Cao, Lin Zou, Guangliang Hao, Zhenyu Zhou, Guichun Zhang

**Affiliations:** Department of Orthopaedics, The General Hospital of Jinan Military, No.25 Shifan Road, Tianqiao Square, Jinan, 250031 China

**Keywords:** Minimally invasive transforaminal lumbar interbody fusion, Unilateral pedicle screw, Bilateral pedicle screw

## Abstract

**Purpose:**

The minimally invasive transforaminal lumbar interbody fusion procedure with percutaneous pedicle screws was adopted in clinical practice, but the choice between a unilateral pedicle screw (UPS) or bilateral pedicle screw (BPS) fixation after lumbar fusion remains controversial. The purpose of the present retrospective study was to compare the clinical outcomes and radiological results of unilateral and bilateral pedicle screw fixations.

**Methods:**

The retrospective study recruited seventy-eight patients with a single-level pedicle screw fixation and lumbar interbody fusion at L4–L5 or L5–S1 from January 2010 to January 2013. The patients were treated with MIS TLIF with BPS fixation, and since May 2012, all patients were treated with UPS fixation. The perioperative outcomes including operative time, blood loss, hospital-stay length, and complication rates were accessed. Radiological outcomes regarding fusion were determined with the Bridwell grading system. Clinical outcomes were evaluated with the Oswestry Disability Index (ODI) and visual analog scale (VAS) during the mean follow-up of 2 years.

**Results:**

According to perioperative assessments, the operative time was significantly shorter for group UPS (84.7 ± 6.4 min) than for group BPS (103.6 ± 10.6 min; *p* < 0.0001), and similar results were found with regard to the mean blood loss (UPS, 96.3 ± 17.5; BPS, 137.4 ± 32.9, *p* < 0.0001). With regard to the hospital-stay period, though the UPS group seems shorter, there is no statistical significance (UPS, 10.0 ± 2.1; BPS, 10.4 ± 2.4, *p* = 0.428). There were four in the BPS group and six in the UPS group defined as unfused at 6 months pest-operative, but at 12 months post-surgery, all patients achieved solid fusion. Regarding clinical outcomes, the VAS and ODI scores were significantly lower in the UPS group than the BPS group at 7 days post-surgery, but there was no difference at 1 month post-surgery and during the later follow-up.

**Conclusion:**

There was no difference between the UPS and BPS flexion techniques about the clinical outcomes at 24 months post-surgery. However, because the UPS involves a shorter surgical time, less blood loss, faster pain relief, and faster functional recovery, UPS might be more suitable in performing single-segment pedicle screw fixation and lumbar interbody fusion.

## Introduction

Transforaminal lumbar interbody fusion (TLIF) is a traditional and popular technique used to treat various lumbar degenerative disorders. Recently, with the progression of modern instrumentations, the minimally invasive transforaminal lumbar interbody fusion (MIS TLIF) procedure with percutaneous pedicle screws was adopted in clinical practice, with the advantages of less approach-related muscle damage, less blood loss, less postoperative pain, shorter length of hospital stay, and also allows for early ambulation [1]. Generally, bilateral pedicle screw (BPS) fixation is accepted as a standard procedure in lumbar interbody fusion. Providing rigid fixation, BPS has great biomechanical stability and several clinical advantages. Recently, for the purpose of being more and more minimally invasive, the unilateral pedicle screw (UPS) fixation has been performed, with an expected effective result as BPS fixation [2–5]. However, as the goal of lumbar interbody fusion is to achieve a solid arthrodesis of spinal segments that can sustain loading [6], the effect of UPS flexion is questionable at times, and controversy continues with the outcomes.

A comparative study with 87 cases of degenerative spondylolisthesis demonstrated no significant differences in clinical outcomes and fusion rates between UPS and BPS for a follow-up period of over 24 months [7]. Only a few clinical studies reported better outcomes of patients with UPS [8–10]. And in an in vivo animal model, Goel et al. showed that UPS was consistently less rigid than BPS [11]. Similarly, a recent in vitro biomechanical study demonstrated that UPS systems have significantly increased segmental range of motion, less stiffness, and increased off-axis movement [12]. The UPS flexion provided only half of the improvement in stiffness compared with the other constructs tested. Similar results were confirmed in a recent finite element analysis [13]. Controversy continues with the effectiveness of the UPS flexion technique. The purpose of the present retrospective study was to compare the clinical outcomes and radiological results of unilateral and bilateral pedicle screw fixation 2 years after surgery.

## Methods

**Fig. 1:**
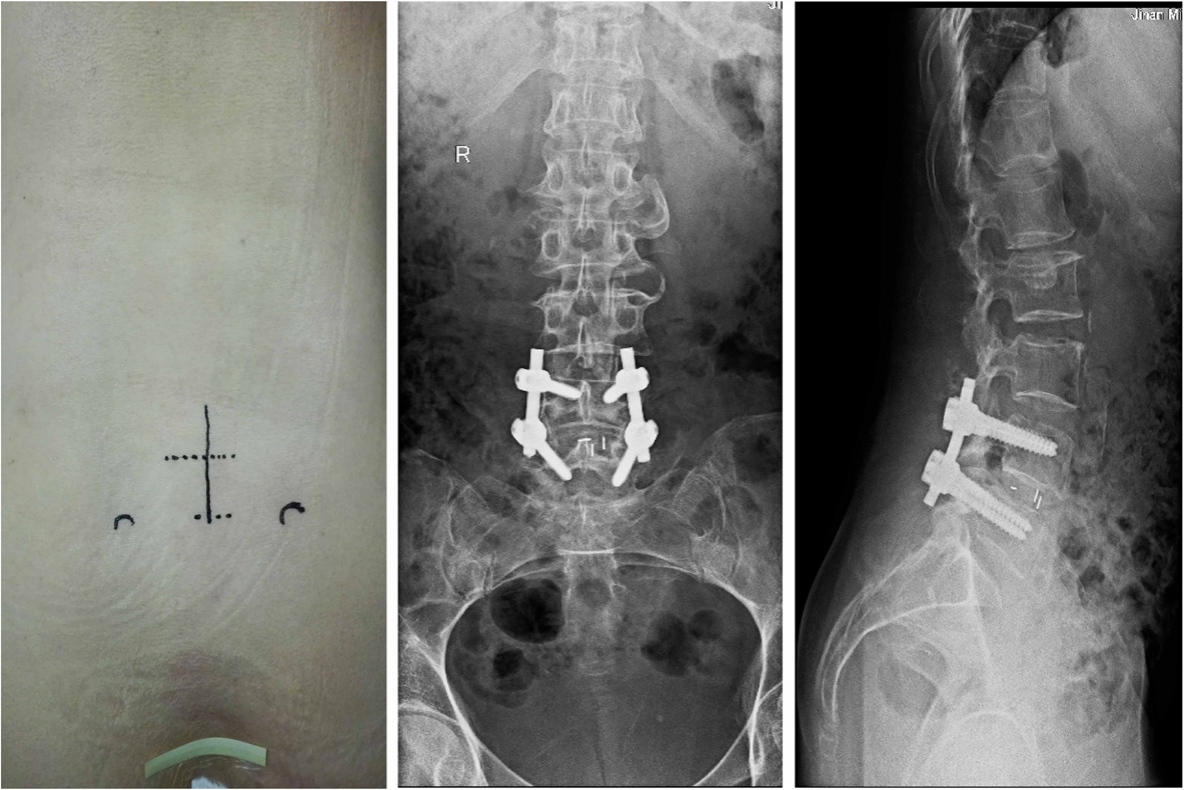
Anteroposterior and lateral CT scan showing MIS TLIF with BPS fixation in lumbar spinal fusion

**Fig. 2:**
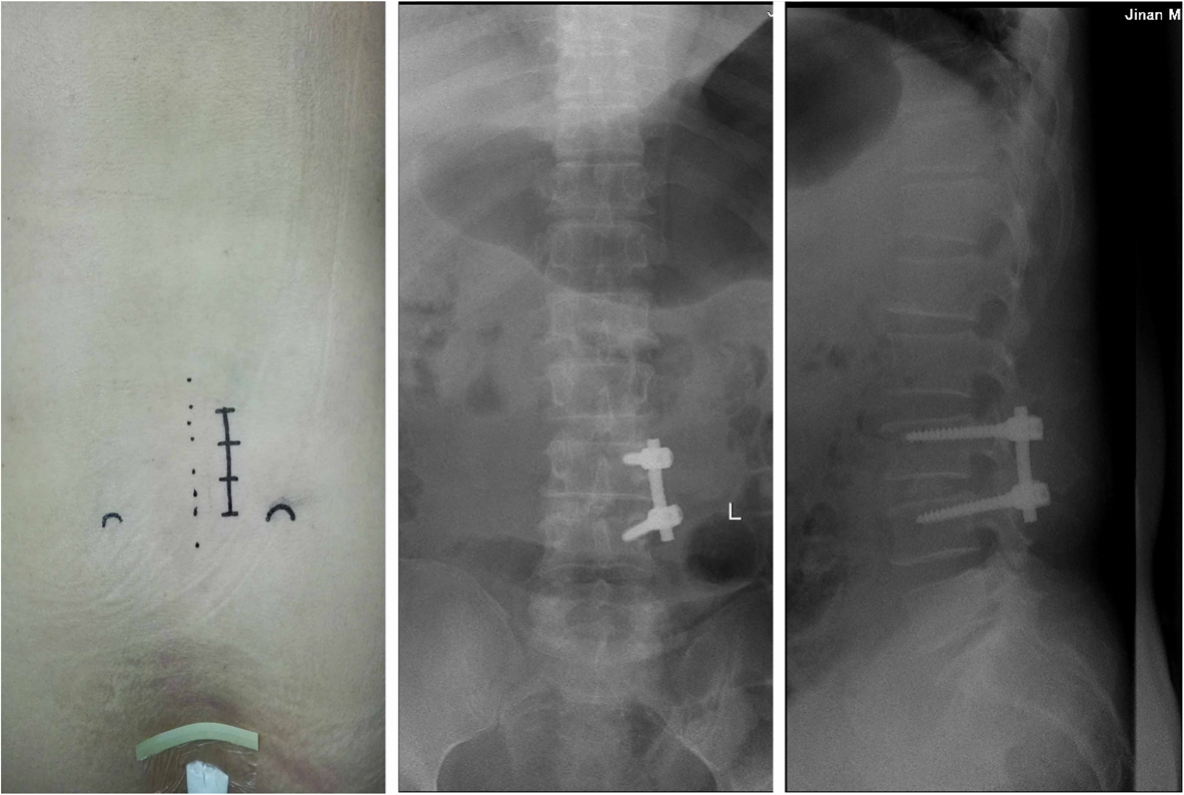
Anteroposterior and lateral CT scan showing MIS TLIF with UPS fixation in lumbar spinal fusion

This project was approved by the Institutional Review Board of the General Hospital of Jinan Military. The present retrospective study recruited 78 patients with the treatment of single-level pedicle screw fixation and lumbar interbody fusion at L4–L5 or L5–S1 from January 2010 to January 2013. All patients had suffered from low back pain, severe unilateral radicular pain, or neurological symptoms. All patients underwent at least 6 months of conservative management before surgery, with no response or an inadequate response. All patients were diagnosed with plain radiographs, CT scans, and MRI. Patients were included if they were aged between 40–70 years and without spondylolisthesis. Patients who were significantly obese (body mass index ≥35 kg/m2), had previous lumbar fusion or discectomy, lumbar tumors, severe osteoporosis, active inflection, RA, or other underlying conditions were excluded from the study. From January 2010 to May 2012, the patients were treated with MIS TLIF with BPS fixation (group A, Fig. 1), and since May 2012, all patients were treated with UPS fixation (group B, Fig. 2). Demographics and procedure data for the two groups are listed in Table 1.

**Table 1:** Comparison of the demographic data of the patients in the BPS and the UPS groups

		BPS	UPS	Total
Number of patients		42	36	78
Age (years)		64 (51–69)	63 (52–70)	64 (51–70)
Sex		29 male	26 male	55 male
		13 female	10 female	23 female
Follow-up (months)		27 (18–36)	24 (19–28)	25 (18–36)
Operative indication	Unilateral lumbar disc herniation	16	13	29
	Foraminal stenosis	22	17	39
	Discogenic low back pain	4	6	10
Level of fusion	L4–L5	18	17	35
	L5–S1	24	19	43

### Surgical approaches

The MIS TLIF procedure was performed on the side that was more symptomatic. After general endotracheal anesthesia, patients were placed in the prone position. C-arm fluoroscopy was used to determine the operative level. A 2.5-cm incision was made, and a MetRx tubular retractor (Medtronic Sofamor Danek, Memphis, TN, USA) was placed. The total facetectomy was performed, which means the isthmus, the posterior arch of the vertebrae, and the inferior joint facet. These bones from osteotomy were kept for autograft during the interbody fusion. The thecal sac and traversing nerve root were identified. Extensive decompression of the contralateral side was performed, including the central stenosis, the ligamentum flavum and its bony attachment, the deep cortical surface of the contralateral lamina, and the contralateral lateral recess and foramen. Then, discectomy and endplate preparation were performed, and the disc space was packed with the autograft bones described above. A PEEK interbody graft (Capstone, Medtronic Sofamor Danek) was then inserted. Finally, with the help of the Sextant system (Medtronic Sofamor Danek), percutaneous lumbar pedicle screws were inserted unilaterally or bilaterally.

### Post-surgery rehabilitation

The patients were recommended to begin ambulation after wearing a lumbus sacrum orthosis (LSO) after at least 7 days of bed rest. The brace will be worn for about 2 months. During the first 7 days, only bed exercises were performed. After 7 days, exercise more than daily life activity was not allowed; in fact, the patients were encouraged to rest more and do exercise less than daily life activity.

### Outcomes

The demographic data including age, sex, preoperative diagnosis, and degenerated segment were searched and recorded from the medical record. The operative time, blood loss, hospital-stay length, and complication rates were accessed. Clinical outcomes were evaluated with the Oswestry Disability Index (ODI) and visual analog scale (VAS) at 7 days; 1 and 6 months; and 1 and 2 years after surgery. Patients underwent CT and radiography at 6 months and 1 year after surgery. Radiological outcomes regarding fusion were determined with the Bridwell grading system. The Bridwell system is composed of the following categories and grades: fused with remodeling and trabeculae present (grade I); graft intact, not fully remodeled and incorporated, but no lucency present (grade II); graft intact, potential lucency present at the top and bottom of the graft (grade III); and fusion absent with collapse/resorption of the graft (grade IV) [14].

### Statistical analysis

The Statistical Package for Social Sciences software (SPSS, Inc., Chicago, IL, USA), version 16.0 for Windows was used for statistical analysis in this study. The clinical data were presented as Mean ± SD and compared between groups by the Student’s *t*-tests. Demographic data and radiological results were assessed with chi-square test. The *p* < 0.05 was considered to indicate a statistically significant difference.

## Results

The patients in the two groups were comparable because there were no significant differences in their ages, genders, and the follow-up period. All of the patients were followed up for a median time of 25 months (range from 18 to 36 months). The operative segments also did not differ significantly between groups. The characteristics of the two groups are shown in Table 1.

According to perioperative assessments, the operative time was significantly shorter for group UPS (84.7 ± 6.4 min) than for group BPS (103.6 ± 10.6 min; *p* < 0.0001), and similar results were found with regard to the mean blood loss (UPS, 96.3 ± 17.5; BPS, 137.4 ± 32.9, *p* < 0.0001). With regard to the hospital-stay period, though the UPS group seems shorter, there is no statistical significance (UPS, 10.0 ± 2.1; BPS, 10.4 ± 2.4, *p* = 0.428). Among all patients, no complications like inflections happened, but there were three cases where the cage moved slightly toward the spinal canal (two with UPS and one with BPS), but no reoperation was performed as the movement induced no discomfort.

The fusion rate was analyzed at 6 and 12 months postoperatively. There were four in the BPS group and six in the UPS group defined as unfused at 6 months pest-operative, but at 12 months post-surgery, all patients achieved solid fusion (Table 2).

**Table 2:** Comparison of the clinical results observed in the patients of the BPS and the UPS groups

		BPS	UPS	*p* value
Operation time (min)		103.6 ± 10.6	84.7 ± 6.4	<0.0001
Blood loss (ml)		137.4 ± 32.9	96.3 ± 17.5	<0.0001
Hospital stay (days)		10.4 ± 2.4	10.0 ± 2.1	0.428
Fusion	6 months	38/42	30/36	0.868
	12 months	42/42	36/36	1

Regarding clinical outcomes, the VAS and ODI scores were significantly lower in the UPS group than the BPS group at 7 days post-surgery, but there was no difference at 1 month post-surgery and the latter (Table 3). Radicular symptoms like numbness/weakness were also compared between the groups. Only three patients in the UPS group and three patients in the BPS group felt numbness of the leg 7 days postoperative, and all of the radiculopathy improved with time as no patient complained about it after 6 months of follow-up.

**Table 3:** Comparison of the VAS and ODI score between the groups

		7 days	1 month	6 months	24 months
Vas	BPS	4.43 ± 1.04	2.38 ± 0.58	2.19 ± 0.67	1.83 ± 0.58
	UPS	3.61 ± 1.52	2.36 ± 0.58	2.25 ± 0.50	2.06 ± 0.41
	*P* value	0.0063	n.s	n.s	n.s
ODI	BPS	18.5 ± 3.92	14.3 ± 2.79	10.9 ± 3.27	7.19 ± 2.77
	UPS	16.1 ± 4.55	14.3 ± 3.39	11.4 ± 2.98	8.03 ± 3.01
	*p* value	0.0168	n.s	n.s	n.s

## Discussion

The most important finding of the study was that there was no difference between the UPS and BPS flexion techniques about the clinical outcomes at 24 months post-surgery. However, because the UPS involves a shorter surgical time, less blood loss, faster pain relief, and faster functional recovery, UPS might be more suitable for performing the single-segment pedicle screw fixation and lumbar interbody fusion.

TLIF requires unilateral total facetectomy, so iatrogenic instability is a possibility, and additional pedicle screw fixation is essential [12, 15], but the choice between unilateral or bilateral pedicle screw fixation after lumbar fusion remains controversial. BPS fixation after lumbar interbody fusion is accepted as a standard procedure, with great biomechanical stability and clinical benefits resulting from rigid fixation. After Goel et al. [11] first reported the benefits of UPS fixation, several clinical trials have also found that unilateral pedicle screw fixation is as effective as bilateral pedicle screw fixation in lumbar spinal fusion [16]. Moreover, as the rigidity of BPS fixation can lead to device-related osteoporosis [17] and makes the adjacent segment prone to load- and motion-induced degeneration [18], the use of less rigid systems of fixation such as UPS flexion has been advocated [19, 20]. Nevertheless, UPS fixation may be detrimental to spine stability and the promotion for fusion as suggested by an in vitro study [12]. Therefore, the use of unilateral or bilateral PS fixation remains a matter of debate.

Numerous previous biomechanical studies have attempted to comparatively evaluate the unilateral and bilateral PS fixation approach, and inconsistent results were also obtained. Chen et al. demonstrated that UPS fixation was good enough to maintain the stability of the spine in a biomechanics study [16]. On one hand, similar studies confirmed that the UPS system was effective to reduce stress shielding of the vertebra and diminish peak stress arising in the adjacent levels above and below the fusion [11]. Moreover, Toyone and coauthors reported that UPS fixation resulted with a lower incidence of adjacent-segment degeneration than BPS fixation. But on the other hand, Aoki et al. observed that UPS fixation caused postoperative cage migration more frequently than BPS fixation [21]. Another study also found that UPS fixation supplied only half of the improvement in stiffness compared with BPS fixation and caused significant off-axis rotational motions, which could hinder stability and the promotion for fusion after TLIF. [12] Yucesoy et al. also reported that UPS fixation was inadequate to stabilize a 2-level unilateral lesion when compared with BPS fixation [22].

Similar with the biomechanical researches, the conclusions of clinical studies are also controversial. A prospective study of 87 patients demonstrated that the UPS fixation was as effective as BPS fixation in lumbar spinal fusion independent of the number of fusion segments (one or two segments) or pedicle screw systems. [7] Similar results reported that UPS instrumented TLIF is a safe, feasible, and viable treatment option generating better results, especially in terms of operative time, blood loss, and hospital time for single-level disease and implant costs. No decrease in the fusion rate or increase in the complication rate was observed during 2 years of follow-up [20, 23–25]. Two-level unilateral instrumented TLIF is an effective and safe method with reduced operative time and blood loss for multiple-level lumbar diseases, but it is imperative that the larger cage should be appropriately positioned to support the contralateral part of the anterior column by crossing the midline of the vertebral body [26]. However, a recently prospective randomized controlled study demonstrated that though UPS fixation with a single-cage technique is effective enough to improve patients’ symptoms and is less invasive than a 2-cage technique and BPS fixation, it resulted in less improvement in back pain, lower-extremity pain, and lower-extremity numbness [27]. Moreover, another study demonstrated that although perioperative results were better with unilateral screw fixation, the long-term results were better with bilateral screw fixation, suggesting that bilateral screw fixation is a better choice after MIS TLIF. [28] The present study demonstrated similar clinical outcomes at the 2-year follow-up and better perioperative outcomes, which are similar with the previous mentioned studies. However, as shown in Table 3, though there were no statistical differences between the groups with regard to the VAS and ODI scores during follow-up, the VAS and ODI scores in the BPS group seems lower than the UPS group, which may suggest a better outcome with BPS fixation for a long-term follow-up.

There are some limitations in this study. The present study is a retrospective study, a prospective randomized controlled study with a larger population will offer a higher level of evidence. In addition, the follow-up period of 2 years was relatively short for detecting long-term outcomes, especially the load- and motion-induced degeneration of the adjacent segment and the device-related osteoporosis. Further studies with longer follow-up and larger study populations are also needed to determine the clinical significance of scoliotic change after UPS fixation with MIS TLIF.

## Conclusion

There was no difference between the UPS and BPS flexion techniques about the clinical outcomes at 24 months post-surgery. However, because the UPS involves a shorter surgical time, less blood loss, faster pain relief, and faster functional recovery, UPS might be more suitable for performing single-segment pedicle screw fixation and lumbar interbody fusion.
